# Prevalence and risk factors of atrioventricular block among 15 million Chinese health examination participants in 2018: a nation-wide cross-sectional study

**DOI:** 10.1186/s12872-021-02105-3

**Published:** 2021-06-11

**Authors:** Ruiqi Shan, Yi Ning, Yuan Ma, Siliang Liu, Jing Wu, Xiaohan Fan, Jun Lv, Bo Wang, Shijun Li, Liming Li

**Affiliations:** 1 Department of Epidemiology and Biostatistics, School of Public Health, Peking University Health Science Center, 38 Xueyuan Road, Beijing, 100191 China; 2 Peking University Health Science Center Meinian Public Health Institute, 35 North Huayuan Road, Beijing, 100191 China; 3 Peking University Center for Public Health and Epidemic Preparedness & Response, Beijing, China; 4 Meinian Institute of Health, Beijing, China; 5 Department of Anesthesiology, The Fourth Affiliated Hospital of Harbin Medical University, Harbin, China; 6 Department of Cardiology, Fuwai Hospital, National Center for Cardiovascular Diseases, Chinese Academy of Medical Sciences and Peking Union Medical College, Beijing, China

**Keywords:** Atrioventricular block, Epidemiology, Prevalence, China, Risk factor

## Abstract

**Background:**

Nationwide data on the prevalence of atrioventricular (AV) block are currently unavailable in China. Thus, we aimed to assess the prevalence and risk factors of AV block among Chinese health examination adults.

**Methods:**

A total of 15,181,402 participants aged ≥ 18 years (mean age 41.5 ± 13.4 years, 53.2% men) who underwent an electrocardiogram as a part of routine health examination in 2018 were analyzed. AV block was diagnosed by physicians using 12-lead electrocardiogram. Overall and stratified prevalence (by age, sex, and city size) of all, first-, second- and third-degree AV block were calculated. Multivariable logistic regression analyses were performed to explore risk factors associated with AV block.

**Results:**

AV block was observed in 88,842 participants, including 86,153 with first-degree, 2249 with second-degree and 440 with third-degree AV block. The age- and sex-standardized prevalence rate [95% confidence interval (CI)] of all, first-, second- and third-degree AV block were 7.06‰ (7.01–7.11), 6.84‰ (6.79–6.89), 0.18‰ (0.17–0.18) and 0.04‰ (0.03–0.04) respectively. After multivariable adjustment, the risk of AV block was positively associated with older age, being male, lower heart rate, higher body mass index, hypertension, diabetes and low high-density lipoprotein cholesterol. High total cholesterol was associated with a lower risk of AV block.

**Conclusion:**

First-degree AV block is relatively common while severe AV block is rare in health examination adults. Besides, AV block was highly prevalent among the elderly. The risk of AV block was associated with older age, being male and metabolic factors.

**Supplementary Information:**

The online version contains supplementary material available at 10.1186/s12872-021-02105-3.

## Introduction

Atrioventricular (AV) block is a loss of normal function of the cardiac conductive pathway between the atrium and the ventricle. It can be divided into three degrees: first-degree, second-degree (type-I or II) and third-degree [1, 2]. The first-degree and type-I second-degree AV block are considered to be relatively benign conditions [1, 2]. However, accumulating evidence suggests that PR interval prolongation, which is the electrocardiograph feature of the first-degree and type-I second-degree AV block, is associated with atrial fibrillation [3], heart failure [4] and mortality [3–5]. Moreover, the prognosis of type-II second-degree and third-degree AV block are generally poor and always require clinical treatment such as medication or pacemaker implantation [6, 7]. Without proper and timely treatment, AV block might lead to death [3–6].

Given the potential serious threats to human health, it is necessary to investigate the early development of AV block. However, to our knowledge, such studies are scarce in China and worldwide, especially among ostensibly healthy population. Only one study investigated the prevalence of first-degree AV block among 10,926 participants in China. Nevertheless, the study conduct among residents aged ≥ 40 years in rural Northeast China [8]. Moreover, no Chinese data on the prevalence of second and third-degree AV block are available among ostensibly healthy population.

In this study, we aimed to examine the prevalence of first-, second- and third-degree AV block and explored related risk factors of AV block based on the database of 15,181,402 Chinese adults from 30 provinces who underwent a 12-lead electrocardiogram (ECG) as a part of routine health examination in 2018.

## Methods

### Study design and participants

This study was conducted using the database of Meinian Healthcare Group, which is the top non-public health examination institution and provides health examination across China. Each health screening centers equipped with professional and experienced medical teams provided comprehensive health examinations for participants.

In the current analysis, we extracted data between January 1st, 2018 and December 31st, 2018, which were derived from 454 centers from 224 cities in 30 provinces. Among 16,988,851 adults aged 18 years or older, after excluding missing value in sex (n = 15) and ECG (n = 1,807,434), 15,181,402 adults (89% of the participants) with routine ECG test were included in the final analysis.

The study was approved by the Peking University Institutional Review Board with a waiver of informed consent (IRB00001052-19077). Data on individual identification were removed and only anonymous information was kept during the study process.

### Assessment of AV block

A twelve-lead standard ECG was performed for each participant. First-degree AV block was defined as abnormal prolongation of the PR interval (greater than 0.2 s) and each atrial excitement is transmitted to the ventricle. Second-degree AV block was defined as atrial impulse partly cannot be transmitted to the ventricular. Third-degree AV block was defined as complete absence of atrioventricular conduction [2]. All ECGs were reading by at least two well-trained cardiologist. In case of controversy, a senior cardiologist was consulted for a final diagnosis or until agreement was reached with discussion.

### Assessment of social demography, anthropometric and laboratory factors

The demographic and clinical history were obtained by health professional through face to face interview. City size was determined according to urban population size. For better understand we reclassified urban population size as follow: more than five million as big city, one to five million as middle city, less than one million as small city [9].

Anthropometric data such as height, weight, blood pressure and heart rate were measured using standard methods. Hypertension was defined as systolic blood pressure ≥ 140 mmHg and/or diastolic blood pressure ≥ 90 mmHg and/or self-reported history of hypertension and/or use of antihypertensive treatment [10]. Body mass index (BMI) was calculated as weight (kg)/height (m)^2^ and was categorized into < 18.5 kg/m^2^ (underweight), 18.5 kg/m^2^ ≤ BMI < 24 kg/m^2^ (normal), 24 kg/m^2^ ≤ BMI < 28 kg/m^2^ (overweight), and ≥ 28 kg/m^2^ (obese), respectively [11].

Blood samples were drawn by venipuncture after 8–12 h of overnight fasting to measure fasting blood glucose (FBG), high-density lipoprotein cholesterol (HDL-C), low-density lipoprotein cholesterol (LDL-C), total cholesterol (TC) and triglyceride (TG). Laboratory measurements were measured with commercially available reagents at the clinical biochemical laboratories in each center. Diabetes was defined as FBG ≥ 7.0 mmol/L and/or self-reported history of diabetes [12]. Low HDL-C was defined HDL-C < 1.0 mmol/L. High LDL-C was defined as LDL-C ≥ 4.1 mmol/L. High TC was defined as TC ≥ 6.2 mmol/L. High TG was defined as TG ≥ 2.3 mmol/L [13].

### Statistical analysis

Characteristics of the participants are presented as No. (%) for categorical variables. Chi square tests were used to compare differences in categorical variables between participants with and without AV block. Age- and sex-standardized prevalence with 95% confidence interval (CI) was calculated according to population of 2010 China Population Sampling Census using direct standardization method. Age-standardized prevalence was calculated for men and women, respectively. Sex-standardized prevalence was calculated for specific age group. Chi square tests was applied to compare rates in different subgroups. Unadjusted, age and sex adjusted, and multivariable logistic regression analyses (adjusting for age, sex, city size, heart rate, BMI, hypertension, diabetes, low HDL-C, high LDL-C, high TC and high TG) were conducted to investigate risk factors for AV block. Curve graph of prevalence rates by different age was drawn using GraphPad Prism 8. Map of prevalence rates across 30 provinces was drawn using R version 4.0.4 (http://www.r-project.org/) to present geographical variation visually. Analyses were conducted using SAS, version 9.3 (SAS Institute, Inc., Cary, North Carolina). *P* < 0.05 was considered statistically significant.

## Results

Among the 15,181,402 study participants, 88,842 participants were diagnosed with AV block, including 86,153 with first-degree, 2249 with second-degree and 440 with third-degree AV block. Characteristics of the participants by AV block status are presented in Table 1. Compared with those without AV block, participants with AV block were more likely to be older, men, with lower heart rate, with higher BMI and living in cities with smaller urban population size, and have a higher proportion of hypertension, diabetes, low HDL-C, high LDL-C, high TC and high TG (Table 1).

**Table 1:** Characteristics of study participants by atrioventricular block status

	Overall	Without AV block	Participants with AV block			
			All	First degree	Second degree	Third degree
N	15,181,402	15,092,560	88,842	86,153	2249	440
Age, years						
18–39	7,609,253 (50.1)	7,588,277 (50.3)	20,976 (23.6)	20,072 (23.3)	784 (34.9)	120 (27.3)
40–59	5,896,375 (38.8)	5,859,173 (38.8)	37,202 (41.9)	36,302 (42.1)	750 (33.3)	150 (34.1)
≥ 60	1,675,774 (11.0)	1,645,110 (10.9)	30,664 (34.5)	29,779 (34.6)	715 (31.8)	170 (38.6)
Sex						
Men	8,079,772 (53.2)	8,013,421 (53.1)	66,351 (74.7)	64,499 (74.9)	1567 (69.7)	285 (64.8)
Women	7,101,630 (46.8)	7,079,139 (46.9)	22,491 (25.3)	21,654 (25.1)	682 (30.3)	155 (35.2)
City size*						
Small city	4,570,409 (30.1)	4,536,248 (30.1)	34,161 (38.5)	33,106 (38.4)	873 (38.8)	182 (41.4)
Middle city	6,094,497 (40.1)	6,058,120 (40.1)	36,377 (40.9)	35,452 (41.2)	773 (34.4)	152 (34.5)
Big city	4,516,496 (29.8)	4,498,192 (29.8)	18,304 (20.6)	17,595 (20.4)	603 (26.8)	106 (24.1)
Heart rate, bpm						
< 60	377,629 (3.4)	372,179 (3.4)	5450 (8.3)	4931 (7.7)	360 (21.7)	159 (50.2)
60–100	10,614,677 (95.6)	10,554,664 (95.6)	60,013 (91.2)	58,570 (91.8)	1285 (77.6)	158 (49.8)
> 100	115,780 (1.0)	115,443 (1.0)	337 (0.5)	326 (0.5)	11 (0.7)	0
BMI, kg/m^2^						
< 18.5	640,013 (4.7)	638,248 (4.7)	1765 (2.2)	1646 (2.1)	92 (4.6)	27 (6.8)
18.5–23.9	6,504,666 (47.6)	6,476,822 (47.7)	27,844 (34.6)	26,731 (34.2)	945 (46.9)	168 (42.3)
24–27.9	4,690,273 (34.3)	4,655,934 (34.3)	34,339 (42.6)	33,456 (42.8)	730 (36.2)	153 (38.5)
≥ 28.0	1,825,423 (13.4)	1,808,789 (13.3)	16,634 (20.6)	16,337 (20.9)	248 (12.3)	49 (12.3)
Hypertension						
No	11,469,363 (78.1)	11,417,566 (78.2)	51,797 (60.3)	50,128 (60.2)	1442 (66.3)	227 (53.5)
Yes	3,209,2182 (21.9)	3,175,183 (21.8)	34,099 (39.7)	33,168 (39.8)	734 (33.7)	197 (46.5)
Diabetes						
No	13,851,682 (94.9)	13,775,997 (95.0)	75,685 (87.9)	73,307 (87.8)	1990 (91.5)	388 (90.2)
Yes	749,368 (5.1)	738,904 (5.1)	10,464 (12.1)	10,237 (12.3)	185 (8.5)	42 (9.8)
Low HDL-C						
No	9,477,347 (91.1)	9,415,791 (91.1)	61,556 (87.7)	59,759 (87.6)	1504 (90)	293 (92.7)
Yes	925,535 (8.9)	916,866 (8.9)	8669 (12.3)	8479 (12.4)	167 (10)	23 (7.3)
High LDL-C						
No	9,700,780 (93.7)	9,635,735 (93.7)	65,045 (92.9)	63,187 (92.9)	1567 (93.9)	291 (92.4)
Yes	651,784 (6.3)	646,837 (6.3)	4947 (7.1)	4822 (7.1)	101 (6.1)	24 (7.6)
High TC						
No	12,305,558 (91.2)	12,229,928 (91.2)	75,630 (90.3)	73,356 (90.2)	1901 (92.2)	373 (91.4)
Yes	1,186,254 (8.8)	1,178,085 (8.8)	8169 (9.7)	7974 (9.8)	160 (7.8)	35 (8.6)
High TG						
No	11,386,284 (84.5)	11,318,687 (84.6)	67,597 (80.8)	65,443 (80.6)	1799 (87.6)	355 (87.4)
Yes	2,082,218 (15.5)	2,066,129 (15.4)	16,089 (19.2)	15,784 (19.4)	254 (12.4)	51 (12.6)

Characteristics of the participants are presented as No. (%) for categorical variables. *P* < 0.001 for all comparisons between participants with and without AV block

*AV* atrioventricular, *BMI* body mass index, *Low HDL-C* high-density lipoprotein cholesterol < 1.0 mmol/L, *High LDL-C* low-density lipoprotein cholesterol ≥ 4.1 mmol/L, *High TC* total cholesterol ≥ 6.2 mmol/L, *High TG* triglyceride ≥ 2.3 mmol/L

*City size was determined according to urban population size

Age- and sex-standardized prevalence (95% CI) for all, first-, second- and third-degree AV block were 7.06‰ (7.01–7.11), 6.84‰ (6.79–6.89), 0.18‰ (0.17–0.18) and 0.04‰ (0.03–0.04), respectively. The prevalence was higher among older participants (age ≥ 60 years vs. age 18–39 years: 18.0 ‰ vs. 2.67‰ for all AV block; 17.5‰ vs. 2.55‰ for first-degree AV block; 0.42‰ vs. 0.10‰ for second-degree AV block; 0.10‰ vs. 0.02‰ for third-degree AV block), higher among men (men vs. women: 10.3‰ vs. 3.92‰ for all AV block; 9.98‰ vs. 3.78‰ for first-degree AV block; 0.25‰ vs. 0.12‰ for second-degree AV block; 0.05‰ vs. 0.03‰ for third-degree AV block), higher among small city (small city vs. big city: 8.02‰ vs. 5.38‰ for all AV block; 7.76‰ vs. 5.18‰ for first-degree AV block; 0.21‰ vs. 0.17‰ for second-degree AV block; 0.05‰ vs. 0.03‰ for third-degree AV block) (Table 2).

**Table 2:** The prevalence of atrioventricular block among Chinese health examination adults in 2018

	All AV block (no. of cases = 88,842)		First degree AV block (no. of cases = 86,153)		Second degree AV block (no. of cases = 2249)		Third degree AV block (no. of cases = 440)	
	Crude prevalence	Standardized prevalence^a^	Crude prevalence	Standardized prevalence^a^	Crude prevalence	Standardized prevalence^a^	Crude prevalence	Standardized prevalence^a^
Total	5.85 (5.81–5.89)	7.06 (7.01–7.11)	5.67 (5.64–5.71)	6.84 (6.79–6.89)	0.15 (0.14–0.15)	0.18 (0.17–0.18)	0.03 (0.03–0.03)	0.04 (0.03–0.04)
Age, years								
18–39	2.76 (2.72–2.79)	2.67 (2.63–2.70)	2.64 (2.60–2.67)	2.55 (2.51–2.59)	0.10 (0.10–0.11)	0.10 (0.09–0.01)	0.02 (0.01–0.02)	0.02 (0.01–0.02)
40–59	6.31 (6.25–6.37)	6.23 (6.16–6.29)	6.16 (6.09–6.22)	6.08 (6.01–6.14)	0.13 (0.12–0.14)	0.13 (0.12–0.14)	0.03 (0.02–0.03)	0.03 (0.02–0.03)
≥ 60	18.3 (18.1–18.5)	18.0 (17.8–18.3)	17.8 (17.6–18.0)	17.5 (17.3–17.7)	0.43 (0.40–0.46)	0.42 (0.39–0.45)	0.10 (0.09–0.12)	0.10 (0.09–0.12)
Sex								
Men	8.21 (8.15–8.27)	10.3 (10.2–10.4)	7.98 (7.92–8.04)	9.98 (9.89–10.1)	0.19 (0.18–0.20)	0.25 (0.24–0.26)	0.04 (0.03–0.04)	0.05 (0.04–0.06)
Women	3.17 (3.13–3.21)	3.92 (3.86–3.98)	3.05 (3.01–3.09)	3.78 (3.72–3.83)	0.10 (0.09–0.10)	0.12 (0.11–0.12)	0.02 (0.02–0.03)	0.03 (0.02–0.03)
City size*								
Small city	7.47 (7.4–7.55)	8.02 (7.92–8.11)	7.24 (7.17–7.32)	7.76 (7.67–7.85)	0.19 (0.18–0.20)	0.21 (0.20–0.23)	0.04 (0.03–0.05)	0.05 (0.04–0.06)
Middle city	5.97 (5.91–6.03)	7.37 (7.29–7.46)	5.82 (5.76–5.88)	7.17 (7.09–7.26)	0.13 (0.12–0.14)	0.17 (0.15–0.18)	0.03 (0.02–0.03)	0.04 (0.03–0.04)
Big city	4.05 (3.99–4.11)	5.38 (5.29–5.47)	3.90 (3.84–3.95)	5.18 (5.10–5.27)	0.13 (0.12–0.14)	0.17 (0.16–0.19)	0.02 (0.02–0.03)	0.03 (0.02–0.03)

Prevalence rates are presented as ‰ (95% confidence interval)

*AV* atrioventricular

*City size was determined according to urban population size

^a^Prevalence rates were standardized for age and sex, prevalence rates in different age group were standardized for sex only, prevalence rates in different sex were standardized for age only.* P* < 0.001 for all comparisons between subgroups

In multivariable-adjusted models, older age (per 10 years increment OR 1.66, 95% CI 1.64–1.67), being male (OR 2.44, 95% CI 2.39–2.49), higher BMI (per 5 kg/m^2^ increment OR 1.26, 95% CI 1.24–1.28), hypertension (OR 1.08, 95% CI 1.06–1.11), diabetes (OR 1.20, 95% CI 1.17–1.23) and low HDL-C (OR 1.19, 95% CI 1.15–1.22) were associated with an increased risk of AV block. Living in big city (OR 0.78, 95% CI 0.76–0.80), higher heart rate (per 10 bpm increment OR 0.69, 95% CI 0.68–0.70) and high TC (OR 0.88, 95% CI 0.85–0.91) were associated with a decreased risk of AV block after multivariable adjustment. *P* < 0.001 for all (Table 3).

**Table 3:** Odds ratios (95% CIs) for associations between risk factors and atrioventricular block

	AV block (cases/N = 88,842/15,181,402)					
	Unadjusted	*P* value	Age and sex adjusted	*P* value	Multivariable adjusted	*P* value
Age, per 10 years increment	1.73 (1.72, 1.74)	< 0.001	1.73 (1.72, 1.74)	< 0.001	1.66 (1.64, 1.67)	< 0.001
Sex						
Women	1.00		1.00		1.00	
Men	2.61 (2.57, 2.65)	< 0.001	2.65 (2.61, 2.69)	< 0.001	2.44 (2.39, 2.49)	< 0.001
City size*						
Small city	1.00		1.00		1.00	
Middle city	0.80 (0.79, 0.81)	< 0.001	0.93 (0.91, 0.94)	< 0.001	1.01 (0.99, 1.03)	0.208
Big city	0.54 (0.53, 0.55)	< 0.001	0.68 (0.67, 0.69)	< 0.001	0.78 (0.76, 0.80)	< 0.001
Heart rate, per 10 bpm increment	0.96 (0.95, 0.96)	< 0.001	0.70 (0.69, 0.70)	< 0.001	0.69 (0.68, 0.70)	< 0.001
BMI, per 5 kg/m^2^ increment	1.53 (1.52, 1.54)	< 0.001	1.26 (1.25, 1.28)	< 0.001	1.26 (1.24, 1.28)	< 0.001
Hypertension	2.37 (2.34, 2.40)	< 0.001	1.10 (1.09, 1.12)	< 0.001	1.08 (1.06, 1.11)	< 0.001
Diabetes	2.58 (2.53, 2.63)	< 0.001	1.23 (1.21, 1.26)	< 0.001	1.20 (1.17, 1.23)	< 0.001
Low HDL-C	1.45 (1.41, 1.48)	< 0.001	1.23 (1.20, 1.26)	< 0.001	1.19 (1.15, 1.22)	< 0.001
High LDL-C	1.13 (1.10, 1.17)	< 0.001	0.91 (0.89, 0.94)	< 0.001	1.01 (0.97, 1.06)	0.505
High TC	1.12 (1.10, 1.15)	< 0.001	0.88 (0.86, 0.90)	< 0.001	0.88 (0.85, 0.91)	< 0.001
High TG	1.30 (1.28, 1.33)	< 0.001	1.05 (1.03, 1.07)	< 0.001	0.98 (0.96, 1.00)	0.086

Results are presented as odds ratios (95% confidence intervals). Multivariable adjusted model adjusted for all co-variables listed in the table

*AV* atrioventricular, *BMI* body mass index, *Low HDL-C* high-density lipoprotein cholesterol < 1.0 mmol/L, *High LDL-C* low-density lipoprotein cholesterol ≥ 4.1 mmol/L, *High TC* total cholesterol ≥ 6.2 mmol/L, *High TG* triglyceride ≥ 2.3 mmol/L

*City size was determined according to urban population size

**Fig. 1:**
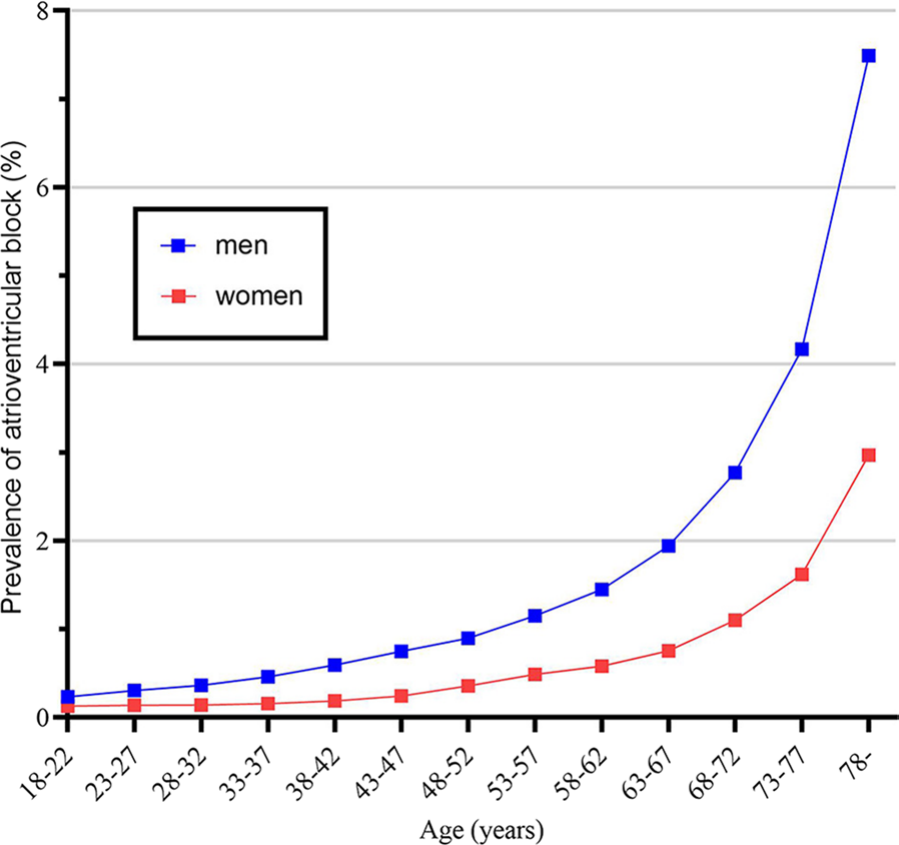
Sex-specific prevalence (%) of atrioventricular block by different age

As shown in Fig. 1 and Additional file 1: Table S1, the prevalence of AV block was lowest in age 18–22 years (men: 0.23%; women: 0.13%) then gradually increased. Notably, the prevalence dramatically increased from age 53–57 years (men: 1.15%; women: 0.48%) and then reached the peak in age ≥ 78 years (men: 7.49%; women: 2.97%).

**Fig. 2:**
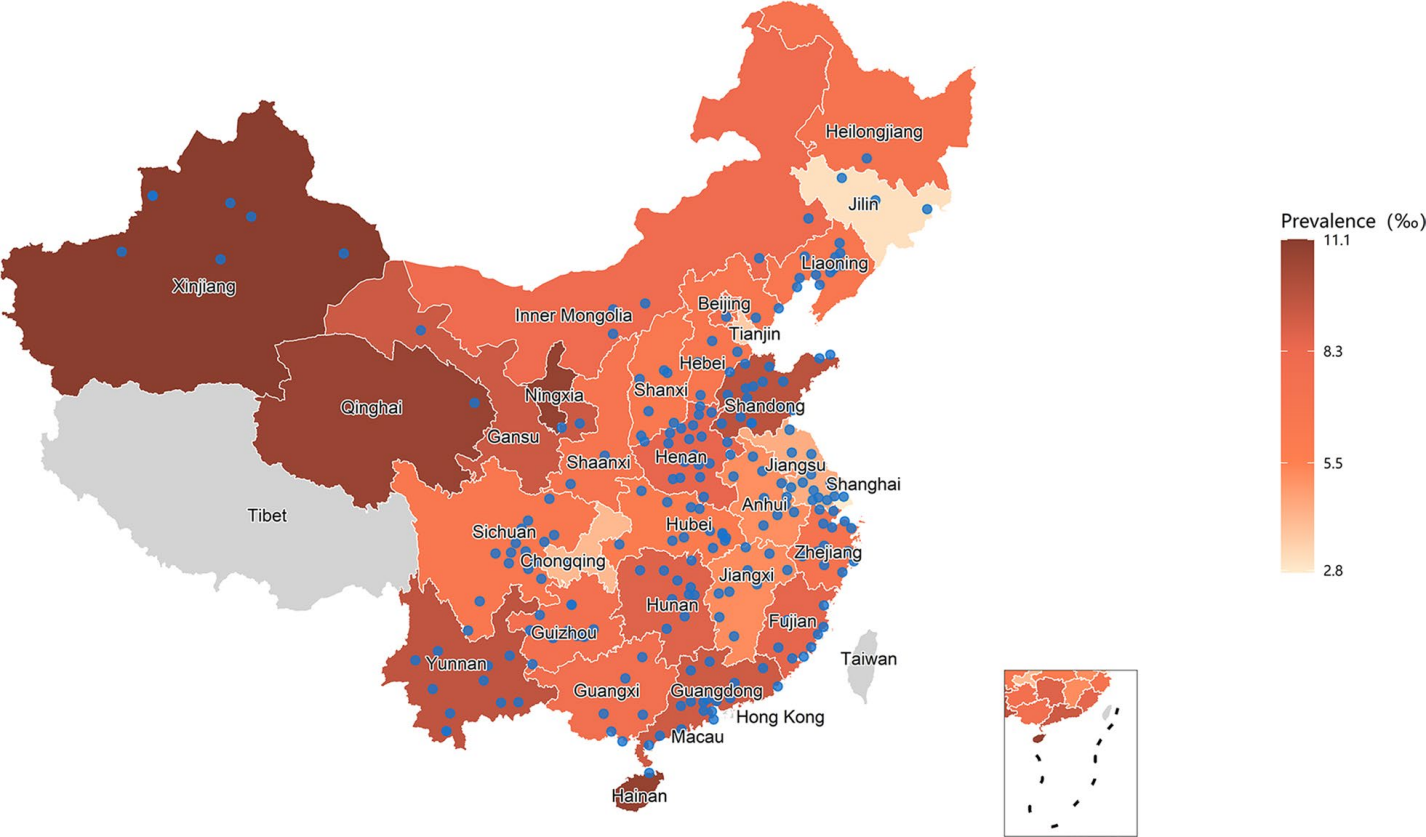
Age- and sex-standardized prevalence of atrioventricular block among Chinese health examination adults in 2018. Data from all province of mainland China were available except Tibet. Blue points represent the locations of 224 cities included in this study

As shown in Fig. 2 and Additional file 1: Table S2, the provinces with age- and sex-standardized prevalence of AV block higher than 10‰ were Xinjiang (11.1‰), Hainan (10.8‰), Ningxia (10.8‰) and Qinghai (10.7‰), respectively. The provinces with age- and sex-standardized prevalence of AV block lower than 4‰ were Shanghai (2.8‰), Jilin (3.1‰) and Tianjin (3.6‰), respectively.

## Discussion

In this study, we reported on the prevalence of all, first-, second- and third-degree AV block among 15 million Chinese adults aged ≥ 18 years who underwent routine physical examination during 2018. The age- and sex-standardized prevalence rates of all, first-, second- and third-degree AV block were 7.06 ‰, 6.84‰, 0.18‰ and 0.04‰, respectively. Further, older age, being male, lower heart rate, higher BMI, hypertension, diabetes and low HDL-C were associated with a higher risk of AV block. Living in big city and high TC were associated with a lower risk of AV block after multivariable adjustment.

To the best of our knowledge, this is the first nationwide study investigating the prevalence of first-, second- and third-degree AV block simultaneously. There is only one study investigated the prevalence of first-degree AV block in China prior to our study. However, this study only focus on the residents aged ≥ 40 years in rural Northeast China [8]. Our study is the first study investigated the prevalence of first-degree AV block among adults aged ≥ 18 years across mainland China (except Tibet). The prevalence of first-degree AV block was reported to be 2% among Finnish general population aged 30–59 years (mean age, 44 years) [5] and 1.6% among Framingham general population aged ≥ 20 years (mean age, 47 years) [3]. Besides, in Asia, it reported to be 1.9% among Japanese general population aged 30–95 years (mean age, 49.9 years) (PR interval greater than 0.22 s) [14] and 3.4% among residents aged ≥ 40 years (mean age, 59.8 years) in rural Northeast China [8]. In our study, the prevalence of first-degree AV block was 0.68% which is relatively lower than those above-mentioned studies. This discrepancy may be partly due to the study participants are younger (aged ≥ 18 years, mean age, 41.5 years) and the majority of participants are ostensibly healthy in the present study. While, the prevalence was relatively close to that reported among United States male flying personnel aged ≥ 16 years (0.65%) [15] and among Swiss conscripts aged 17–38 years (0.76%) [16]. To the best of our knowledge, no epidemiology studies have been conducted on the prevalence of second-degree AV block in China and it was reported to be 0.018% in our study. The prevalence of second-degree AV block was 0.007% among Swiss conscripts aged 17–38 years [16] and 0.006% among United States male flying personnel aged ≥ 16 years [15]. The prevalence of second-degree AV block in these studies are lower than the prevalence in our study, which may be partly due to the variation in age, race and physical fitness (the level of physical fitness may be better among conscripts and fliers). Without prompt and appropriate treatments, third-degree AV block could be fatal. Given the relatively poor prognosis, however, very little is known about the prevalence of third-degree AV block among ostensibly healthy Chinese and it was 0.004% in our study. It was lower than the prevalence reported in the Michigan general population 0.02–0.04% [17] and Reykjavik general population 0.04% [18]. These two studies [17, 18] including participants with obviously severe diseases which are relatively rare in health examination population. And the prevalence of third-degree AV block in our study was little higher than the prevalence among apparently healthy male flying personnel (0.002%) [15], partly owing to the the level of physical fitness may be better among male flying personnel than health examination population.

In our study, older age and being male were significantly associated with higher risk of AV block after multivariable adjustment. These results are in agreement with previous studies [8, 19, 20]. Further, living in small city was associated with higher risk of AV block after multivariable adjustment. That can be attributed to small city (with smaller urban population) may be relevant to a combination of lower socioeconomic status, inadequate health expenditures, and insufficient medical and public health services [21]. Lower heart rate was associated with higher risk of AV block which is attributed to the pathophysiology of AV block [1]. Besides, higher BMI was also associated with higher risk of AV block in our study. That may be because obesity is strongly associated with cardiac fibrosis [22] which is the potential etiology of AV block [1].

Furthermore, hypertension and diabetes were significantly associated with higher risk of AV block. Previous studies have also found that hypertension and diabetes were associated with AV block [19, 23]. Several reasons may provide possible clues for the association between hypertension, diabetes and AV block: Hypertension and diabetes are known predispositions to myocardial infarction [24] and other cardiovascular diseases [25]. Besides, myocardial fibrosis, as a result of long-standing hypertension, may infiltrate AV conduction system, which is speculated to be a possible reason of AV block [26–28]. Additionally, metabolic derangements in diabetic cardiomyocyte could induce inflammation and fibrosis caused by cardiomyocyte injury and cell death [29]. Moreover, low HDL-C has also been observed to associate with higher risk of AV block in our study, partly because HDL-C can protect endothelial cells from inflammation and oxidative stress [30]. Furthermore, serum TC level was associated with lower risk of AV block. Only two population-based studies have investigated the association between TC and AV block and no significant correlation was found, in which the sample size is far smaller than our study [8, 19]. However, plenty of researches have reported the inverse association between TC and atrial fibrillation [31–33]. Cholesterol is a prominent component of membranes and are known to affect properties of cell electrophysiology, which may be implicated in the etiology of cardiac arrhythmias [34, 35]. Further, cholesterol may have a beneficial effect on the immune system and could protect against infections [36]. Nevertheless, the mechanisms underlying the inverse association between TC and AV block have not been defined. Further researches are warranted.

### Strengths and limitations

To our knowledge, this is the first nationwide epidemiological study on the prevalence of first-, second- and third-degree AV block and related risk factors undertaken in China and this is also the first study assessed the prevalence of AV block not only among elderly but also among younger Chinese adults. Moreover, this study based on 15,181,402 Chinese adults covering almost all geographic areas in China, which are various in socioeconomic and geographic features, providing important information for allocating healthcare resources appropriately. Nevertheless, several limitations should be mentioned. First, this was a research based on physical examination population, most of them were ostensibly healthy and without severe clinical symptoms, the generalizability of the results to general population should be made with caution. Secondly, due to our study results are limited by cross-sectional design, further researches are warranted to verify our conclusion on the potential risk factors. Thirdly, information on education level, diet and lifestyle were not available in our studies, which restricted our ability to explore the underlying related risk factors.

## Conclusions

First-degree AV block are most frequently found, second- and third-degree are relatively rare among ostensibly healthy participants. The prevalence of AV block increased significantly with age. Managing metabolic risk factors may be beneficial for preventing AV block. Despite the limitation mentioned before, this study provides unique and valuable evidence for the future population prevention strategies on AV block.

## Supplementary Information

**Additional file 1.** Prevalence of atrioventricular block by age groups and by provinces.

## Data Availability

The datasets used and/or analysed during the current study are available from the corresponding author on reasonable request.

## Abbreviations

AV block: Atrioventricular block
ECG: Electrocardiogram
BMI: Body mass index
FBG: Fasting blood glucose
HDL-C: High-density lipoprotein cholesterol
LDL-C: Low-density lipoprotein cholesterol
TC: Total cholesterol
TG: Triglyceride
CI: Confidence interval
