# Supplementary material for: Prevalence and risk factors of atrioventricular block among 15 million Chinese health examination participants in 2018: a nation-wide cross-sectional study
Source: BMC Cardiovasc Disord. 2021 Jun 11;21:289. doi: 10.1186/s12872-021-02105-3 (PMC8194203; doi:10.1186/s12872-021-02105-3)
Supplement: Supplementary file 1 — Additional file 1. Prevalence of atrioventricular block by age groups and by provinces. [file 12872_2021_2105_MOESM1_ESM.docx]

Supplement Table 1: Prevalence of atrioventricular block by age groups

|  | Prevalence % (95% confidence intervals) | |
| --- | --- | --- |
| Age, years | Men | Women |
| 18-22 | 0.23 (0.22,0.25) | 0.13 (0.11,0.14) |
| 23-27 | 0.31 (0.29,0.32) | 0.14 (0.13,0.15) |
| 28-32 | 0.36 (0.35,0.37) | 0.14 (0.14,0.15) |
| 33-37 | 0.46 (0.45,0.47) | 0.16 (0.15,0.17) |
| 38-42 | 0.59 (0.58,0.61) | 0.19 (0.18,0.20) |
| 43-47 | 0.75 (0.73,0.77) | 0.24 (0.23,0.25) |
| 48-52 | 0.90 (0.88,0.92) | 0.36 (0.34,0.37) |
| 53-57 | 1.15 (1.12,1.17) | 0.49 (0.47,0.51) |
| 58-62 | 1.45 (1.42,1.49) | 0.58 (0.56,0.61) |
| 63-67 | 1.94 (1.89,1.99) | 0.75 (0.72,0.79) |
| 68-72 | 2.77 (2.69,2.85) | 1.10 (1.05,1.16) |
| 73-77 | 4.17 (4.03,4.31) | 1.62 (1.52,1.72) |
| ≥78 | 7.49 (7.30,7.68) | 2.97 (2.82,3.13) |

Supplement Table 2. Prevalence of atrioventricular block by province

| Province | Prevalence % (95% confidence intervals) |
| --- | --- |
| Anhui | 5.14(4.89,5.38) |
| Beijing | 7.24(6.90,7.58) |
| Heilongjiang | 7.06(6.46,7.66) |
| Hubei | 6.00(5.78,6.23) |
| Hunan | 8.71(8.38,9.04) |
| Jilin | 3.05(2.78,3.31) |
| Jiangsu | 4.36(4.14,4.58) |
| Jiangxi | 5.18(4.86,5.50) |
| Liaoning | 6.57(6.39,6.76) |
| Inner Mongolia | 8.06(7.57,8.55) |
| Ningxia | 10.8(9.30,12.3) |
| Qinghai | 10.7(9.32,12.1) |
| Fujian | 8.52(8.07,8.97) |
| Shandong | 9.68(9.47,9.89) |
| Shanxi | 6.39(6.08,6.69) |
| Shaanxi | 6.82(6.52,7.11) |
| Shanghai | 2.75(2.63,2.88) |
| Sichuan | 6.43(6.25,6.61) |
| Tianjin | 3.57(3.29,3.84) |
| Xinjiang | 11.1(10.6,11.7) |
| Yunnan | 9.59(9.25,9.93) |
| Zhejiang | 6.96(6.65,7.27) |
| Chongqing | 4.05(3.68,4.43) |
| Gansu | 9.24(8.74,9.73) |
| Guangdong | 9.18(8.96,9.40) |
| Guangxi | 7.59(7.16,8.02) |
| Guizhou | 7.33(6.90,7.76) |
| Hainan | 10.8(10.1,11.6) |
| Hebei | 6.58(6.27,6.88) |
| Henan | 8.48(8.28,8.67) |

Prevalence rates were standardized for age and sex according to population of 2010

China Population Sampling Census using direct standardization method.
